# Severe Disabling Myalgia as an Initial Presentation of Polyarteritis Nodosa

**DOI:** 10.1155/2019/4364289

**Published:** 2019-04-07

**Authors:** H. M. Senarathna, C. L. Fonseka, H. A. S. Perera, P. U. T. De Silva, T. P. Weerarathna

**Affiliations:** ^1^Registrar in Medicine, University Medical Unit, Teaching Hospital, Karapitiya, Sri Lanka; ^2^Consultant Physician, Department of Internal Medicine, Faculty of Medicine, University of Ruhuna, Galle, Sri Lanka; ^3^Consultant Histo-Pathologist, Department of Pathology, Teaching Hospital, Karapitiya, Sri Lanka; ^4^Senior Registrar in Medicine, University Medical Unit, Teaching Hospital, Karapitiya, Sri Lanka; ^5^Professor in Medicine, Department of Internal Medicine, Faculty of Medicine, University of Ruhuna, Galle, Sri Lanka

## Abstract

**Background:**

Polyarteritis nodosa (PAN) is a form of necrotizing vasculitis affecting medium or small blood vessels with multiorgan involvement. Although myalgia is a clinical feature of PAN, severe disabling myalgia as the initial presentation is rarely noted.

**Case Presentation:**

We present a case of 54-year-old male with recently detected chronic kidney disease admitted with progressive severe disabling muscular pains predominantly over calves with constitutional symptoms for seven weeks. He was weak to mobilize out of the bed. Later, he developed a vasculitic rash, unilateral ulnar claw, and bilateral foot drop. His skin and muscle biopsies showed evidence of vasculitis. His renal and mesenteric artery CT angiogram revealed stenosed segment of the celiac artery without evidence of visible aneurysms elsewhere. He completed six cycles of intravenous cyclophosphamide pulse therapy with high-dose oral prednisolone with good response. With continuation of aggressive immunosuppression and rehabilitation for five months, the patient improved and was able to walk without support.

**Conclusion:**

Musculoskeletal predominant PAN, even though rare, needs to be considered in patients presenting with disabling muscle pain and weakness. These features may herald over days to months along with constitutional symptoms before other systems getting affected. Early recognition of such symptoms and initiating specific treatment would be important for better outcomes.

## 1. Introduction

Vasculitis is characterized by inflammatory cell infiltration and necrosis of vessel walls. This process could affect any organ in the body causing a multisystemic illness. Vasculitis are categorized into large, medium, or small vessel vasculitis depending on the size of the vessel involved and may occur as a primary disease or secondary to infection, malignancy, connective tissue disorders, drugs, etc. [1].

Polyarteritis nodosa (PAN) is a form of necrotizing vasculitis affecting medium or small blood vessels without glomerular nephritis or vasculitis involving arterioles, venules, or capillaries. Affected patients present with nonspecific constitutional symptoms like low-grade fever, malaise, arthralgia, myalgia, loss of appetite, and loss of weight and have organ involvement of the skin, peripheral nervous system, gastrointestinal tract, kidneys, and joints. Investigations may reveal normochromic normocytic anemia with high inflammatory markers but auto-antibodies like antinuclear antibody (ANA), rheumatoid factor (RF), and antineutrophil cytoplasmic antibodies (ANCA) are commonly absent [2]. CT and MRI angiograms will support diagnosis, demonstrating aneurysms or stenosis of medium-sized muscular arteries especially renal and mesenteric vasculature [3]. Treatment options available are high-dose glucocorticoids and immunosuppressants including cyclophosphamide and azathioprine [4].

We describe a patient with PAN who had an unusual muscle involvement. He presented with severe disabling myalgia for several weeks without other manifestations initially, but later diagnosed due to appearance of vasculitic rash and polyneuropathy.

## 2. Case Presentation

A 54-year-old male with chronic kidney disease who was followed up in the nephrology clinic for several months presented with constitutional symptoms, progressive severe pain over both calves, and ankle swelling for seven weeks' duration. The patient was unable to walk due to severe myalgia and was confined to the bed due to worsening pain even with slightest movement. He also had recent arthralgia for which he was seen by a rheumatologist and was under investigation for a seronegative polyarthritis. He had numbness over both feet, progressing over 3 months. On examination, there was significant tenderness over most muscles in the body but predominantly over calf muscles and had worsening pitting ankle edema. He had normal blood pressure and no rashes on the body. There was a stocking type sensory loss over bilateral feet up to the ankles. Rest of the examination was unremarkable.

Initial investigations revealed neutrophil leukocytosis (WBC 14,500/*µ*L) with elevated inflammatory markers of erythrocyte sedimentary rate (ESR) of 120 mm/hr (<25 mm/hr) and C-reactive protein 135 mg/L (<6 mg/L). Hemoglobin was 10.7 g/dl with a normocytic normochromic anemia, and platelets were 298 × 10^9^/l. Blood cultures and urine culture were negative. Urine full report did not reveal red cells or proteinuria. The liver biochemistry panel was within normal range except for serum albumin which was around 24 g/l. Serum creatinine was elevated from his baseline value to 208 *µ*mol/L (baseline around 150 *µ*mol/l–eGFR 31 mL/min/1.73 m^2^). Ultrasonography of abdomen showed small echogenic kidneys which is compatible with his renal condition and showed normal hepatic sonography. His fasting plasma glucose, lipid profile, thyroid function tests, chest radiograph, and electrocardiography were normal.

**Figure 1:**
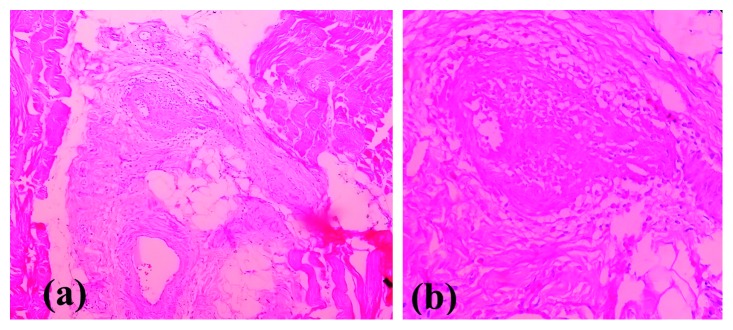
Fibrinoid necrosis of the wall of interfascicular medium vessels with infiltration of inflammatory cells in muscle biopsy: (a) 10 × 10 and (b) 40 × 10.

Due to the disabling muscle aches, we initially performed an ultrasonography which showed some evidence of myositis (muscle inflammation) with no evidence of deep vein thrombosis. Serum level of creatine kinase (CK) is 137 U/L (200–500 U/L), and nerve conduction study/electromyography did not show any evidence of myopathy or myositis. Calf muscle biopsy was performed, and histology showed fibrinoid necrosis of the wall of interfascicular small and medium vessels with infiltration of inflammatory cells (Figure 1).

**Figure 2:**
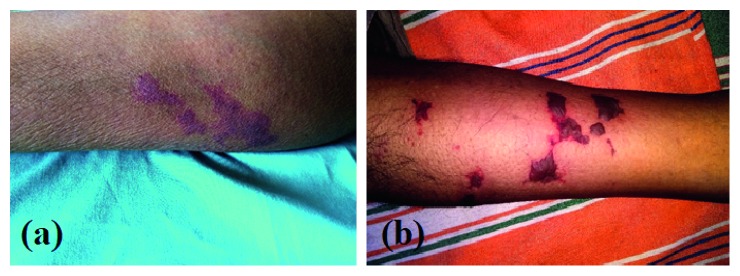
Vasculitic rash over the legs (a), which later developed into the ecchymotic stage (b).

On the day 14 after admission, a vasculitic-type generalized rash appeared over the extensor surfaces of the forearms and legs (Figure 2(a)). Possible vasculitis was suspected, and high-dose steroid was initiated after sending investigations for ANCA, ANA, RF, cryoglobulins, and complements level which were negative. Skin biopsy was performed and sent for the histological evaluation. It demonstrated evidence of acute bullous vasculitis with leukocytoclasia in medium-sized vessels of dermis subsequently. There was no evidence of dysplasia or malignancy. With steroids, the fever and myalgia subsided and the ESR and CRP levels lowered to 32 mm/hr and 18 mg/L, respectively. Subsequently, his blood pressure was found to be elevated at 160/100 mmHg. 2D Echocardiogram, hepatitis B surface antigen, hepatitis C antibody, and HIV screenings were negative, and serum protein electrophoresis did not reveal any monoclonal band.

**Figure 3:**
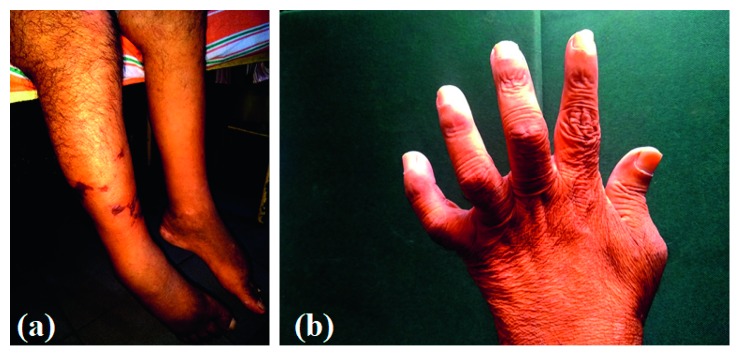
Bilateral foot drop (a) and left ulnar claw (b).

**Figure 4:**
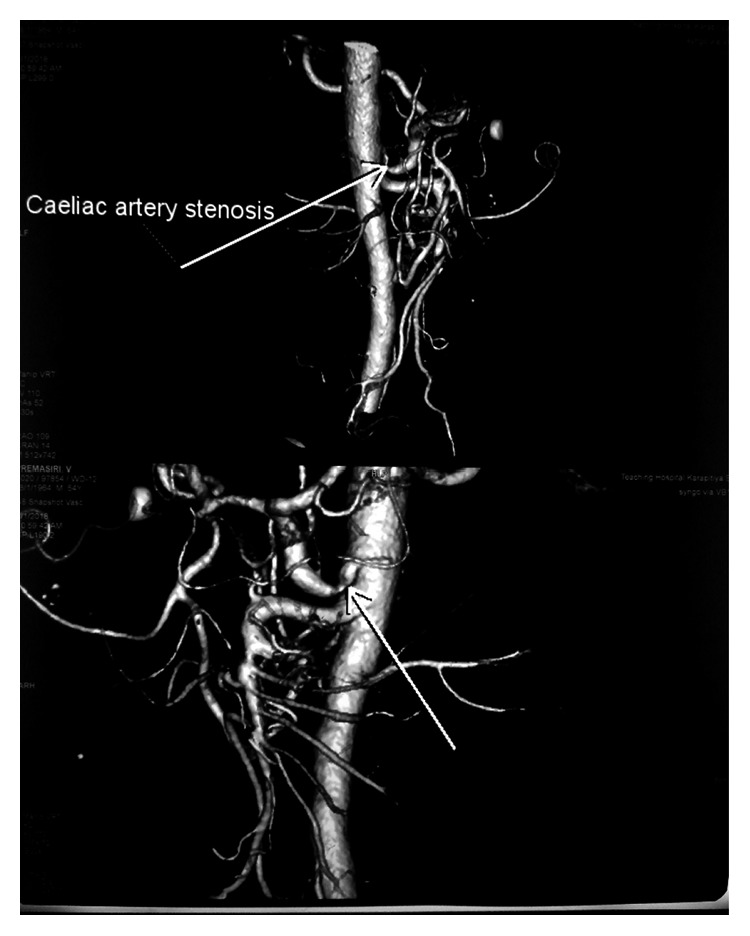
Stenosed segment of the celiac artery in CT angiogram (arrows).

Despite high-dose steroids with pulse therapy over one week, the rash progressed to an ecchymotic stage (Figure 2(b)). Subsequently, we noticed that he developed bilateral foot drop, and after few days, he developed left ulnar claw (Figures 3(a) and 3(b)). Due to the presence of vasculitic rash, worsening polyneuropathy leading to significant disability and renal involvement, we suspected PAN. Hence, we performed a renal and mesenteric artery CT angiogram which revealed 8 mm stenosed segment of celiac artery 5 cm distal to its origin without evidence of visible aneurysms elsewhere (Figure 4). Absent sensory and motor responses were noted for the left ulnar nerve on neurophysiology study which was performed after one month from appearing of left ulnar claw. Due to the worsening nature of the disease, we initiated him on two weekly pulses of intravenous cyclophosphamide and continued high-dose prednisolone for a month and was gradually tailed off.

He completed six cycles of intravenous cyclophosphamide pulse therapy with oral prednisolone. With these medications and rehabilitation, the patient was able to walk without support, almost after five months of treatment. Foot drop and ulnar claw were persistent but with improvement over time.

## 3. Discussion

It was evident that our patients had severe myalgia with constitutional symptoms over several weeks before developing progressive polyneuropathy, hypertension, and vasculitic rash over subsequent weeks. His myalgia was so severe that there was a demonstrable pseudoweakness leading to misinterpretation of the disease as a muscle-related one. His inflammatory markers were persistently elevated with evidence of medium vessel involvement in muscle biopsy and in skin biopsy with evidence of fibrinoid necrosis and also had segmental celiac artery stenosis on angiography. In his case, aggressive immunosuppression was justifiable due to the extent of progressive disability. Though the patient did not complain of a significant weight loss, he has actually lost 10 kilograms over 6 weeks of hospital stay.

Musculoskeletal manifestation of PAN, as myalgia, muscle tenderness, and weakness are well documented, along with other clinical features. Usually, lower limb muscle groups are affected more than upper limbs. Pagnoux et al. [5] have described that 58.6% patients had complained myalgia at some point in their course of illness. Lhote et al. [6] also have described in their review that myalgia was observed as frequent as 30–70% in PAN. But the duration between onset of myalgia and diagnosis of PAN is uncertain. Though previous studies have showed that musculoskeletal manifestation is common, presentation of PAN with predominant initial muscle involvement is rare in the literature.

Nakamura et al. [7] have reported a case of PAN secondary to hepatitis B, which had been presented with acute onset debilitating calf pain over 72 hours with lower limb swelling. In contrast to our patient, involvement of other organs was not evident during the course of illness. They also have analyzed ten case reports of biopsy proven, isolated PAN confined to calf muscles [8–15]. All of them were middle-aged patients whose heralding symptom was calf pain which varied from two to six months. Pain over calf muscles were symmetrical and rapidly responded to steroids except in one which presented with unilateral calf pain and took seven months to respond to steroid [13]. They have not had other system involvement than muscles. But, our patient had extensive multisystem involvement later other than muscles though the patient initially present with predominant musculoskeletal manifestation.

Plumley et al. [16] have summarized seventeen cases in which muscle involvement was the predominant clinical finding as well as there was a noticeable paucity of systemic organ manifestations, very similar to our case. Out of them, sixteen patients presented with muscle pain involving lower limbs, and characteristically, the majority had calf muscle involvement. Only one patient had involvement of upper limb muscles [17]. All of them had a dramatic response to oral corticosteroids or aspirin, without the need for other immunosuppressive agents. Our patient had poor resolution of muscle pains to steroid but responded to more aggressive immunosuppression.

It was evident that there are reports that some patients had longer duration of muscle pains. Ahmed et al. [18] have published a case of PAN which presented with progressive right calf pain and swelling over ten-month duration with overlying skin induration. Another report [19] published a case of 59-year-old Japanese who has presented with prolong pyrexia of undetermined origin and developed calf pain later. Muscle biopsy of gastrocnemius has showed evidence of vasculitis, and he was diagnosed to have PAN confined to calf muscles. PAN has presented with other unusual muscle involvement as well. A recent article [20] described a similar case who presented with diplopia and severe myalgias of the lower extremities with weakness, later diagnosed to have PAN which well responded to high-dose oral steroid.

Rarely, muscular predominant PAN can present as myositis or rhabdomyolysis. Plumley et al. [16] and Iida et al. [21] reported two cases of PAN which presented as polymyositis and rhabdomyolysis, respectively, with very high CPK level. Usually in PAN, the CPK level should be within the normal range. But, slightly elevated CPK in PAN is also mentioned in the literature [7, 16, 21]. Fort et al. [22] reviewed 7 series that included 251 patients, and only 5 (2%) of them had elevated CPK level. It has been hypothesized that fasciitis without involvement of muscle fibers may be the cause of severe muscle pain without elevation of CPK in PAN, vasculitis is usually detected in the fascia, and the normal architecture of muscle fibers is preserved in muscle biopsy [7]. Our patient did not have elevation of CPK, but the literature mentions that PAN should not be excluded depending on high CPK since severe muscle pain is a dominant feature of PAN.

## 4. Conclusions

Musculoskeletal predominant PAN, even though rare, needs to be considered in patients presenting with disabling muscle pain, weakness, or tenderness. These features may herald over days to months along with constitutional symptoms before other systems get affected. Early recognition of such patients and starting specific treatments are important as involvement of the multisystem in PAN carries poor prognosis.

## References

[1] Suresh E. (2006). Diagnostic approach to patients with suspected vasculitis. *Postgraduate Medical Journal*.

[2] Jennette J. C., Falk R. J., Bacon P. A. (2013). 2012 revised International Chapel Hill Consensus Conference Nomenclature of Vasculitides. *Arthritis & Rheumatism*.

[3] Stanson A. W., Friese J. L., Johnson C. M. (2001). Polyarteritis nodosa: spectrum of angiographic findings. *Radiographics*.

[4] James J., Jose J., Thulaseedharan N. K. (2018). Acute necrotizing vasculitic neuropathy due to polyarteritis nodosa. *Oman Medical Journal*.

[5] Pagnoux C., Seror R., Henegar C. (2010). Clinical features and outcomes in 348 patients with polyarteritis nodosa: a systematic retrospective study of patients diagnosed between 1963 and 2005 and entered into the French Vasculitis Study Group Database. *Arthritis & Rheumatism*.

[6] Lhote F., Cohen P., Guillevin L. (1998). Polyarteritis nodosa, microscopic polyangiitis and Churg-Strauss syndrome. *Lupus*.

[7] Nakamura T., Tomoda K., Yamamura Y., Tsukano M., Honda I., Iyama K.-I. (2003). Polyarteritis nodosa limited to calf muscles: a case report and review of the literature. *Clinical Rheumatology*.

[8] Golding D. N. (1970). Polyarteritis presenting with leg pains. *BMJ*.

[9] Leib E. S., Restivo C., Paulus H. E. (1979). Immunosuppressive and corticosteroid therapy of polyarteritis nodosa. *American Journal of Medicine*.

[10] Laitinen O., Haltia M., Lähdevirta J. (1982). Polyarthritis confined to lower extremities. *Scandinavian Journal of Rheumatology*.

[11] Ferreiro J. E., Saldana M. J., Azevedo S. J. (1986). Polyarteritis manifesting as calf myositis and fever. *American Journal of Medicine*.

[12] García F., Pedrol E., Casademont J. (1992). Polyarteritis nodosa confined to calf muscles. *Journal of Rheumatology*.

[13] Gardner G. C. (1993). Polyartreritis nodosa confined to calf muscles. *Journal of Rheumatology*.

[14] Iwamasa K., Komori H., Niiya Y (2001). A case of polyarteritis nodosa limited to both calves with a low titer of MPO-ANCA. *Rheumatism*.

[15] Hall C., Mongey A. B. (2001). Unusual presentation of polyarteritis nodosa. *Journal of Rheumatology*.

[16] Plumley S. G., Rubio R., Alasfar S., Jasin H. E. (2002). Polyarteritis nodosa presenting as polymyositis. *Seminars in Arthritis and Rheumatism*.

[17] Nakauchi Y., Suehiro T., Kumon Y. (1994). Localized polyarteritis nodosa in the forearm and epididymis. *Internal Medicine*.

[18] Ahmed S., Kitchen J., Hamilton S., Brett F., Kane D. (2011). A case of polyarteritis nodosa limited to the right calf muscles, fascia, and skin: a case report. *Journal of Medical Case Reports*.

[19] Miteva M., Norgauer J., Ziemer M. (2007). Diplopia and myalgia. *American Journal of Clinical Dermatology*.

[20] Kamimura T., Hatakeyama M., Torigoe K. (2005). Muscular polyarteritis nodosa as a cause of fever of undetermined origin: a case report and review of the literature. *Rheumatology International*.

[21] Iida H., Hanaoka H., Asari Y. (2018). Rhabdomyolysis in a patient with polyarteritis nodosa. *Internal Medicine*.

[22] Fort J. G., Griffin R., Tahmoush A., Abruzzo J. L. (1994). Muscle involvement in polyarteritis nodosa: report of a patient presenting clinically as polymyositis and review of the literature. *Journal of Rheumatology*.

